# Minding the Gaps in Cancer Pain Management Education: A Multicenter Study of Clinical Residents and Fellows in a Low- Versus High-Resource Setting

**DOI:** 10.1200/JGO.2015.003004

**Published:** 2016-03-23

**Authors:** Charles Amoatey Odonkor, Ernest Osei-Bonsu, Oswald Tetteh, Andy Haig, Robert Samuel Mayer, Thomas J. Smith

**Affiliations:** **Charles Amoatey Odonkor**, **Robert Samuel Mayer**, and **Thomas J. Smith**, Johns Hopkins University School of Medicine, Baltimore, MD; **Ernest Osei-Bonsu**, Komfo Anokye Teaching Hospital, Kumasi; and **Oswald Tetteh**, Korle-Bu Teaching Hospital, Accra, Ghana; and **Andy Haig**, University of Michigan-Ann Arbor, Ann Arbor, MI.

## Abstract

**Purpose:**

Inadequate pain management training has been reported as a major cause of undertreatment of cancer pain. Yet, past research has not comprehensively compared the quality of cancer pain management education among physicians in training in high-resource countries (HRCs) with those in low-resource countries (LRCs). The purpose of this study was to examine and compare gaps in cancer pain management education among physician trainees in an HRC (United States) versus an LRC (Ghana).

**Methods:**

A cross section of physicians at four major academic medical centers completed surveys about the adequacy of cancer pain training. Participation in the study was completely voluntary, and paper or online surveys were completed anonymously.

**Results:**

The response rate was 60% (N = 120). Major gaps were identified in cancer pain management education across the spectrum of medical school training. Training was rated as inadequate (by approximately 80% of trainees), although approximately 10% more trainees in HRCs versus LRCs felt this way; 35% said residency training was inadequate in both settings; and 50% in LRCs versus 44% in HRCs said fellowship training was less than good. On the basis of the lowest group means, the three key areas of perceived deficits included interventional pain procedures (2.34 ± 1.12), palliative care interventions (2.39 ± 1.12), and managing procedural and postoperative pain (2.94 ± 0.97), with significant differences in the distribution of deficits in 15 cancer-pain competencies between LRCs and HRCs (*P* < .05).

**Conclusion:**

This study identifies priority areas that could be targeted synergistically by LRCs and HRCs to advance cancer care globally. The findings underscore differential opportunities to broaden and improve competencies in cancer pain management via exchange training, in which physicians from HRCs spend time in LRCs and vice versa.

## INTRODUCTION

Of an estimated 9 million incident cases of cancer each year, more than 50% are in resource-limited countries, 60% have untreated cancer pain, and 70% are diagnosed at advanced stages of malignancy.^1-3^ Although several international bodies have declared pain relief a human right, a large number of patients in developing countries suffer with cancer pain, have poor quality of life, and often die without any palliative interventions.^4,5^ In several resource-poor settings, this dire situation is exacerbated by a dearth of oncologist, palliative care, and other pain-management specialists.^1,6^

Prior reports indicate that there is little to no active incorporation of pain management in the training curricula for health professionals in developing countries because prevention and treatment of infectious diseases appears to take precedence over pain control.^1,2^ A survey by the International Association for the Study of Pain indicates that few clinicians received adequate training in pain management as undergraduates, and 91% of all respondents stated that a lack of education was the major barrier to pain management in their local health environment.^1,7^ In resource-rich countries such as the United States, more than 50% of patients with cancer have moderate to severe pain that is inadequately treated.^8,9^ Studies in the United States suggest that cancer pain is rarely addressed during medical school training, and although some residency programs may discuss cancer pain management, this happens inconsistently.^7,10,11^

Together, the evidence underscores major gaps in cancer pain management training in both high- and low-resource settings, with a plausible impact on the quality of life of patients with cancer. Yet, to our knowledge, previous research has not comprehensively compared the adequacy and quality of cancer pain management education among physicians in training in high-resource countries (HRCs) versus low-resource countries (LRCs).^12,13^ Moreover, specific deficits in cancer pain management training have yet to be delineated for trainees in low- versus high-resource settings. The objective of this study was to examine the gaps in cancer pain management education among trainees in an LRC (Ghana) versus an HRC (United States). As a result of their broader accessibility to pain management specialties (oncology, physiatry, anesthesiology, and palliative care), we anticipated that trainees in HRCs would report greater competencies beyond the use of opioid analgesics for cancer pain and fewer deficits in specialized cancer pain management procedures and interventions than would their colleagues in LRCs. Identifying deficits in cancer pain management education in high- versus low-resource settings is a necessary step toward designing programs to better equip physicians to provide high-quality care for patients with cancer. By comparing cancer pain education at major academic medical centers in two contrasting resource environments, findings from this study could potentially advance our understanding of the links between resource milieu and barriers to cancer pain education and management.

## METHODS

### Setting

Institutional review board exemption was obtained before study initiation because the study did not qualify as human subject research. A multicenter cross-sectional survey of trainees at four major academic medical centers (two in the United States and two in Ghana) was conducted. All four sites were primarily teaching hospitals with affiliated medical schools. Korle-Bu Teaching Hospital (2,000 beds) and Komfo-Anokye Teaching Hospital (1,200 beds), the two largest hospitals in Ghana, train physicians across all of West Africa, and each hospital has its own oncology department. Johns Hopkins Hospital (1,059 beds) and University Hospital of Michigan–Ann Arbor (1,000 beds) each has its own comprehensive cancer center, and both are part of the National Comprehensive Cancer Network.^14^ Trainees in specialties with expertise in pain management, including physiatry, anesthesiology, and oncology (medical, surgical, and radiation oncology) were selected at random for survey participation. Paper and online questionnaires were sent to physicians to evaluate the educational and training experience caring for patients with cancer-related pain. All surveys were completed anonymously over a 5-month period (July to November, 2015), and participation was completely voluntary, with no incentives offered for survey completion.

### Measures

**Fig 1:**
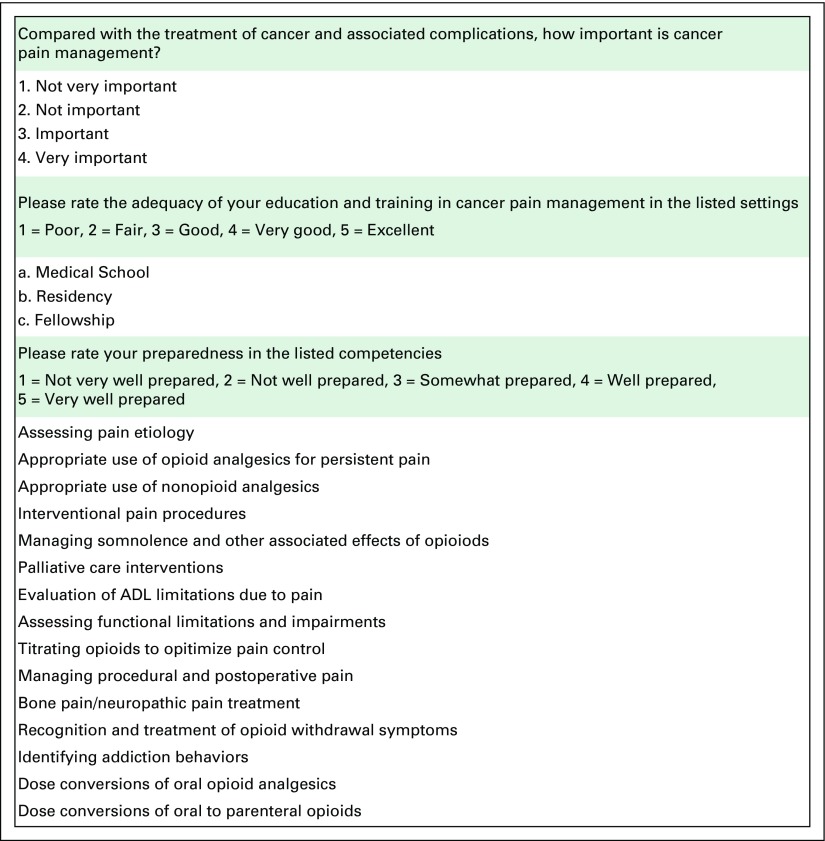
Survey items assessing the perceived importance and adequacy of cancer pain management and training. ADL, activities of daily living.

Questionnaires were adapted from existing pain surveys previously published in the literature and were designed to evaluate the perceived value of cancer pain management education, adequacy of training, and areas of perceived deficits.^10,11^ Survey items are shown in Figure 1. The survey assessed trainees’ impressions of the following domains: importance of treating cancer pain compared with other complications of cancer (responses on a Likert scale of 1 = not very important to 4 = very important); adequacy of education and training in cancer pain management in medical school, residency, and fellowship (responses on a Likert scale of 1 = poor to 5 = excellent); and preparedness in 15 competency areas (responses on a Likert scale of 1 = not very well prepared to 5 = very well prepared).

### Data Analysis

Survey responses were stratified into two groups, high resource (United States) and low resource (Ghana), for comparative analysis of trainee experience in cancer pain management. Descriptive statistics were calculated using nonparametric tests where non-normally distributed data were presented as the median (interquartile range [IQR]) and were compared with the Wilcoxon rank sum test for unpaired data. The percentage of the distribution of responses among trainees was assessed using χ^2^ analysis and Fisher’s exact test. Group means for categorical variables with two levels and more than two levels were assessed with Wilcoxon-Mann-Whitney and Kruskal-Wallis exact tests, respectively. Spearman rank correlation coefficient was used to analyze correlations of training year with perceived value of cancer pain management; ratings of adequacy of training in medical school, residency, and fellowship; and perceived cancer pain management competencies. Competency areas were ranked as high (top three), moderate, and low (bottom three) on the basis of overall group means for each competency area. All statistical analyses were conducted with SAS software (version 9.3; SAS Institute, Cary, NC) using a two-sided hypothesis test, with the probability of a type I error set at .05.

## RESULTS

Of all eligible participants, the response rate was 60% (N = 120). Respondents’ demographic characteristics are listed in Table 1. The median (IQR) age was 31 (IQR, 30-33) and 30 (IQR, 28-33) years for respondents from HRCs and LRCs, respectively. Of 61 respondents from LRCs, 30 were male and 31 were female. Of 59 respondents from HRCs, 30 were female and 29 were male. Compared with LRCs, respondents from HRCs were mostly in physiatry (34 of 59) and anesthesiology (25 of 59). There were 46 residents (78%), 10 fellows (16.9%), and three specialists (5.1%) in the high-resource group, whereas 49 residents (80.3%) and 12 fellows (19.7%) comprised the respondents from the low-resource group. Few respondents from the high-resource group were in the first year of training (1.7%), whereas respondents from the low-resource group comprised 16 trainees (26.2%) in the first year (eleventh month of training).

**Table 1:**
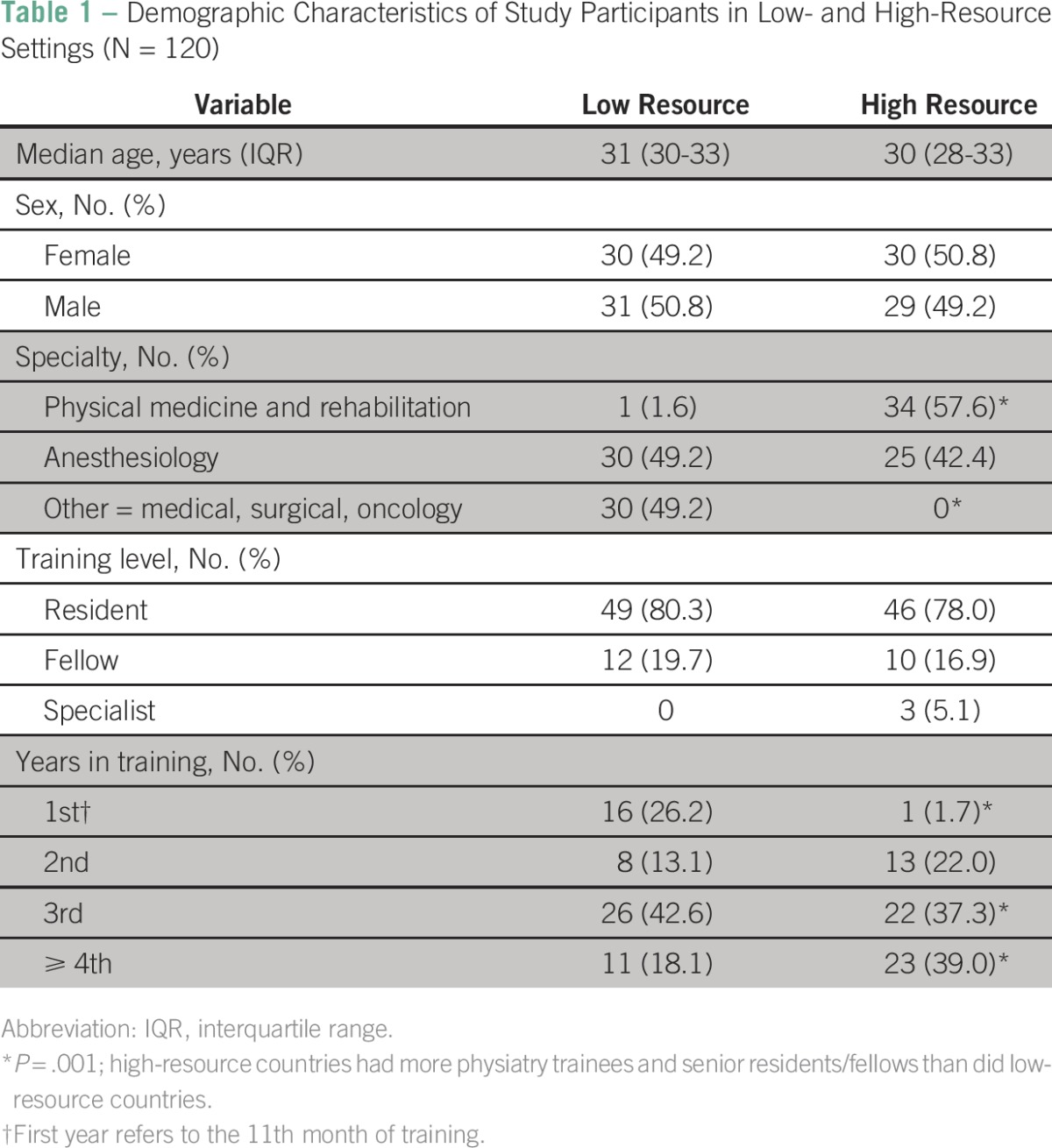
Demographic Characteristics of Study Participants in Low- and High-Resource Settings (N = 120)

### Importance of Cancer Pain Management

**Fig 2:**
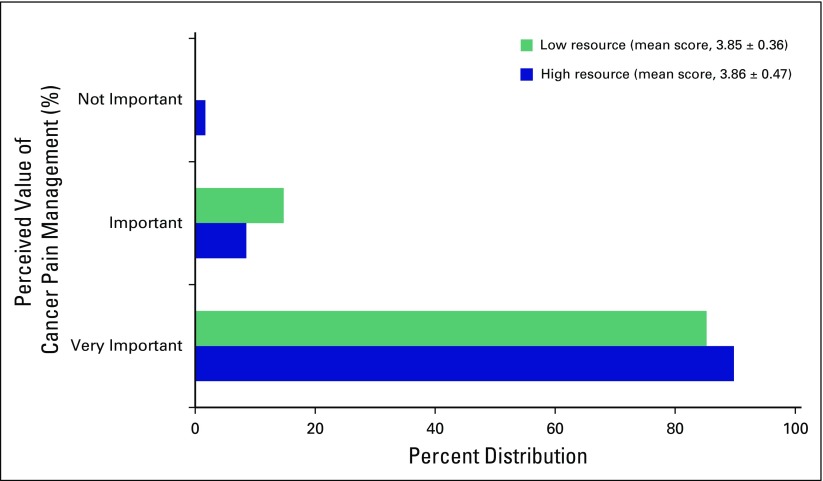
Ratings of the importance of cancer pain management compared with treating other sequelae of cancer. Responses were rated on a Likert scale of 1 (not very important) to 4 (very important).

Ratings of the perceived importance of cancer pain management are summarized in Fig 2. The difference in mean ratings between trainees in LRCs (3.85 ± 0.36) and HRCs (3.86 ± 0.47) did not reach statistical significance (*P* = .4). Overall, however, the majority of trainees felt that cancer pain management was either very important (90% in HRCs and 85% in LRCs) or important (8.5% in HRCs and 14.8% LRCs). A small minority (1.7% in HRCs and 0% LRCs) felt cancer pain management was not important.

### Adequacy of Cancer Pain Management Education and Training

**Fig 3:**
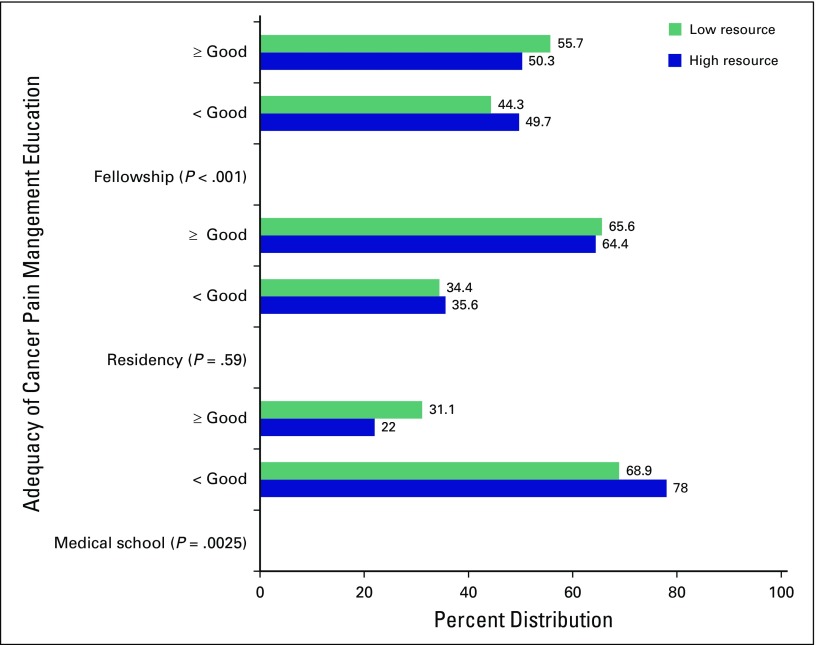
Ratings of the adequacy of cancer pain management education in low- versus high-resource settings. Residents and fellows were asked to rate their experiences during medical school, residency, and fellowship years. “Not applicable” was included as an option so that, for example, residents could select not applicable if rating the fellowship experience. Responses were rated on a Likert scale of 1 (poor) to 5 (excellent). For analysis, responses were categorized into greater than or equal to good versus less than good, where < good = (fair + poor) and ≥ good = (good + very good + excellent).

Ratings of the perceived adequacy of cancer pain management education and training are presented in Figure 3. The ratings among trainees in LRCs versus HRCs varied significantly by the educational and training setting (*P* < .05). In medical school, more trainees in HRCs (78%) than in LRCs (68.9%) felt that cancer pain management training and education were inadequate (less than good). The rest of HRC (22%) versus LRC (31.1%) respondents rated their medical school training as adequate (good or greater than good; *P* = .0025).

There was no significant difference in ratings of residency training in cancer pain management between residents in LRCs and HRCs (*P* = .59). Most trainees thought residency training was good or greater than good (64.4 in HRCs *v* 65.6 in LRCs) compared with those who thought it was less than good (35.6 in HRCs *v* 34.4 in LRCs). About half of trainees in LRCs (49.7%) thought fellowship training in cancer pain management was inadequate versus 44.3% of HRC trainees. The majority of HRC trainees (55.7%) felt their training was good or greater than good.

The training year was positively correlated with perceived adequacy of fellowship training in cancer pain management (Spearman *r* = 0.23, *P* = .01) but not with ratings of residency training (*P* = .90). There was no statistically significant correlation between ratings of perceived importance of cancer pain management and adequacy of training and education in cancer pain management (*P* = .15).

### Perceived Deficits in Cancer Pain Management Competencies

Specific areas of perceived deficits in cancer pain management training are listed in Table 2. On the basis of the lowest group means, the three key areas of perceived deficits were interventional pain procedures (2.34 ± 1.12), palliative care interventions (2.39 ± 1.12), and managing procedural and postoperative pain (2.94 ± 0.97). Areas where trainees felt most competent included appropriate use of opioid analgesics for persistent pain (4.23 ± 0.75), assessing pain etiology (3.73 ± 0.73), and dose conversions of oral opioid analgesics (3.63 ± 0.85). *Z* scores were obtained to compare the differences in mean scores of low- versus high-resource groups with the total group mean. Trainees in low- versus high-resource settings differed most significantly in the following competencies: assessing functional limitations and impairments (*z* = 3.90, *P* < .001), identifying addiction behaviors (*z* = 3.96, *P* < .001), and dose conversions of oral to parenteral opioids (*z* = 5.41, *P* < .001).

**Table 2:**
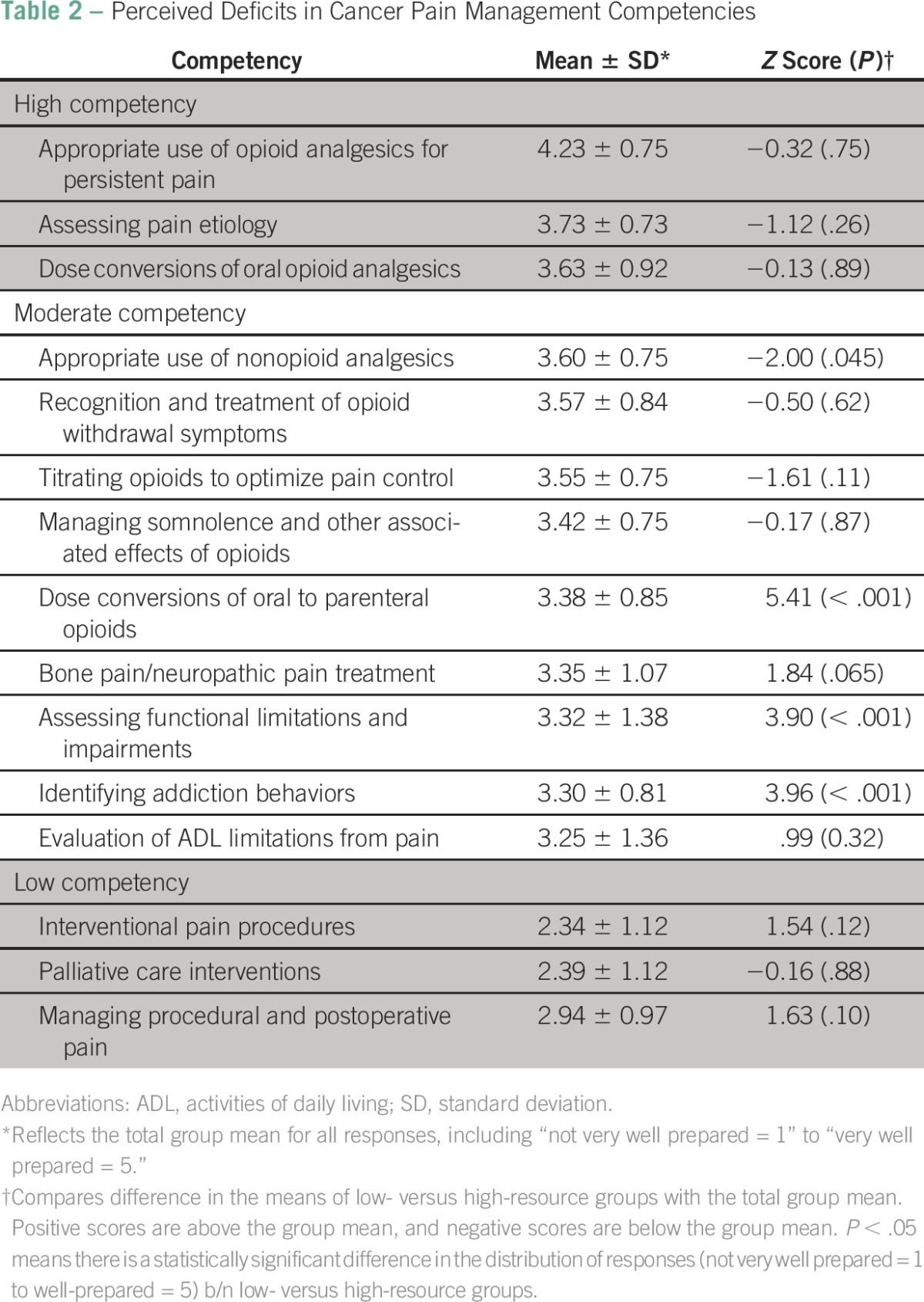
Perceived Deficits in Cancer Pain Management Competencies

### Distribution of Perceived Deficits Among Low- Versus High-Resource Trainees

For more granular analysis of self-perceived deficits beyond the mean and *z* scores, the total percentage of those who felt less than well prepared among each group (low- *v* high-resource) was computed as the percent of less than well-prepared (= not very well prepared + not well prepared + somewhat prepared)/total number of respondents within specified resource category (Table 3). Significant differences in trainees’ perceived deficits were identified in all areas except for bone pain/neuropathic pain treatment (42.4% in HRCs *v* 59.1% in LRCs; *P* = .09) and recognition/treatment of opioid withdrawal symptoms (49.2% in HRCs *v* 45.9% in LRCs; *P* = .95).

**Table 3:**
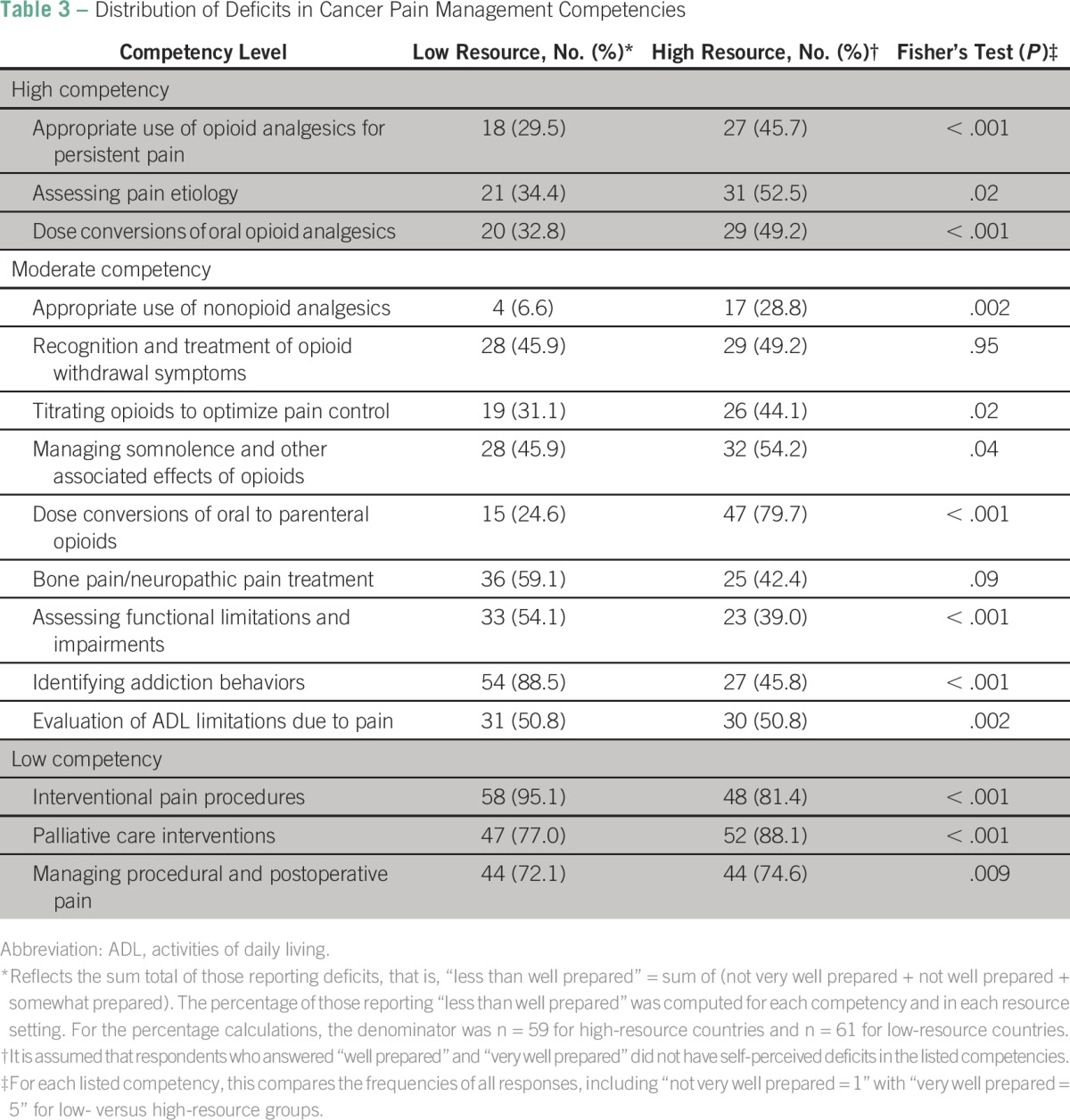
Distribution of Deficits in Cancer Pain Management Competencies

For the top three competency areas where trainees felt most competent, the breakdown was as follows: appropriate use of opioid analgesics for persistent pain (45.7% in HRCs *v* 29.5% in LRCs; *P* < .001), assessing pain etiology (52.5% in HRCs *v* 34.4% in LRCs; *P* = .02), and dose conversions of oral opioid analgesics (49.2% in HRCs *v* 32.8% in LRCs; *P* < .001; Table 3). For the bottom three competencies, the breakdown was as follows: interventional pain procedures (81.4% in HRCs *v* 95.1% in LRCs; *P* < .001), palliative care interventions (88.1% in HRCs *v*. 77% in LRCs; *P* = .001), and managing procedural and postoperative pain (74.6% in HRCs *v* 72.1% in LRCs; *P* = .009). For moderate competency ranking, notable differences were observed in the following areas: assessing functional limitations and impairments (39% in HRCs *v* 54.1% in LRCs; *P* < .001), identifying addiction behaviors (45.8% in HRCs *v* 88.5% in LRCs; *P* < .001), and dose conversions of oral to parenteral opioids (79.7% in HRCs *v* 24.6% in LRCs; *P* < .001). The rest of the breakdown is listed in Table 3.

## DISCUSSION

This study is unique in evaluating the perceived value, adequacy, and quality of cancer pain management education and deficits in pain competencies among trainees in low- versus high-resource settings at four major academic medical centers in North America and sub-Saharan Africa. The first major finding of this study is that most trainees considered treatment of cancer-related pain as important or very important compared with treating other sequelae of cancer, regardless of resource setting. The second major finding is that trainees in low- versus high-resource settings differed slightly in their impression of the adequacy of cancer pain management training across the spectrum of medical training; medical school training was generally rated as inadequate (approximately 80% of trainees), although about 10% more trainees in high- versus low-resource settings felt this way (Fig 3). Trainees were more aligned in their rating of cancer pain education in residency (approximately 34% in both high- and low-resource settings rated cancer pain training as inadequate). There was less agreement regarding fellowship training, with about 50% in LRCs versus 44% in HRCs rating fellowship training as inadequate. This finding expands on prior studies, which show that oncologists and other medical specialists who manage cancer pain have significant knowledge deficiencies in cancer pain management and the need for continued pain management education even among those who are experts.^11,15^ Our work highlights major gaps in cancer pain management education across the spectrum of medical training in both HRCs and LRCs. The third major finding of this study is that the bottom three areas where most trainees felt the least competent were in the following competencies: interventional pain procedures, palliative care interventions, and managing procedural and postoperative pain.

Although it was expected that fewer trainees in high-resource settings compared with their low-resource colleagues would report deficits in advanced cancer pain interventions, it was surprising that approximately 10% more high-resource trainees reported deficits in palliative care (88.1% of high-resource trainees *v* 77% of low-resource trainees; *P* < .001). This discrepancy could be because more trainees in LRCs may be exposed to advanced stages of cancer requiring palliation rather than curative interventions, in part a result of late rather than early cancer diagnosis in resource-limited settings. As indicated in a recent World Health Organization report, often the best course of action for patients in LRCs is pain relief and palliation rather than curative measures.^16^ In a global atlas of palliative care released in 2014, the United States was classified as category 4b (countries with advanced integration of palliative care into mainstream service provision), whereas Ghana was classified in the low category of 3a (countries with patchy and poor palliative care provision).^6^ That a majority of trainees in these distinct environments report deficits in palliative care training underscores differential opportunities to improve the status quo in both countries.^17^

For HRCs, involving trainees in the early phase of cancer care may not only enhance experiential learning of upstream palliative interventions to improve patient quality of life, mood, and survival,^18^ but it could also help them develop an essential competency in cancer pain management. For LRCs, palliative care could be incorporated into primary care services because a bulk of care is provided by general practitioners in the absence of specialists.^1,2^ There is also an opportunity for innovative use of telehealth to enhance cancer pain management education and to increase trainee competence.^19^ A plausible reason that fewer trainees in HRCs felt they had low competence in interventional pain procedures and managing procedural and postoperative pain may be because they had more years of training (about 39% of HRC trainees *v* 18% of LRC trainees had at least 4 or more years of training). This aligns with the correlation results showing that training year was positively correlated with perceived adequacy of fellowship training in cancer pain management (Spearman *r* = 0.23; *P* = .01). It also underscores a role for specialized training to acquire highly technical interventional pain management skills. Because training year correlated with perceived competency, senior trainees and specialists who are likely to have more cancer pain management experience could participate in teaching their junior colleagues.

Collaborative training and educational programs between LRCs and HRCs may help target the other identified deficits, including assessing functional limitations and impairments, identifying addiction behaviors, dose conversions of oral to parenteral opioids, and appropriate use of opioid versus nonopioid analgesics. Physicians in LRCs may share lessons on cost-effective cancer pain management measures, whereas those from HRCs may share expertise in advanced pain management techniques. Because pain in the late stages of cancer is more likely to be due to the malignancy itself and less likely to treatment complications,^6,17^ opportunities for exchange training in which physicians from HRCs spend time in LRCs and vice versa could prove instructive in broadening trainee exposure and improving competencies in cancer pain management.

Despite the guidelines in the World Health Organization’s analgesic ladder, cancer pain remains uncontrolled in resource-limited settings, partly because of a lack of opioid availability, which makes using the ladder approach to pain control rather difficult, if not impossible.^2,20-22^ A recent report indicates that HRCs disproportionately account for the increase in global opioid consumption.^23^ The rates of opioid use in LRCs for cancer pain remains negligible because of legal restrictions, especially in sub-Saharan Africa.^23^ Ghana remains one of the few countries in Africa where there are no opioid prescription form restrictions, and any legitimate pharmacy in the country may dispense opioids.^23^ In a global study examining actual and formulary availability of opioids and costs to patients, morphine equivalents (immediate- and controlled-release oral morphine and injectable morphine) are available and free to all patients in Ghana.^23,24^ Compared with its cohort of LRCs, Ghana is far ahead of its peers. Although legal restrictions remain a valid concern in some LRCs, the fact that the strongest opioids are widely and readily available in Ghana for health care practitioners to use in patient care makes for an intriguing study.^23,24^ This allows for fair comparisons with the United States, an HRC, in regard to how the legal environment may influence trainee experience and competency in cancer pain management. Researchers seeking to untangle the web of factors influencing LRCs’ versus HRCs’ trainee education in oncologic pain care should consider evaluating the impact of national policies on opioid use and regulation on cancer pain management. In addition to cancer pain education, other measures, such as investments in palliative care and integration into national health systems and addressing the cultural stigma associated with cancer, are potential helpful ways to augment cancer pain treatment and outcomes.^23,24^

Overall, this study is, to our knowledge, the first to delineate a comparative list of perceived deficits in major cancer pain management competencies among trainees in two different health and resource environments. There is ample opportunity for LRCs to contribute globally to new developments in cancer pain management strategies, especially because LRCs carry a disproportionate burden of newly diagnosed cases.^25,26^ HRCs investing in capacity building in LRCs could benefit from reverse innovation arising from clinical trials conducted in resource-poor environments.^27-29^ This article provides priority areas that could be targeted synergistically by LRCs and HRCs to advance cancer care globally. In addition to addressing the identified competencies, other systemic barriers not directly assessed in this study warrant consideration by clinicians interested in improving cancer pain management education and patient outcomes.

This study has some limitations. For one, the results must be considered within the context that only a cross section of trainees at four academic medical centers was surveyed. Therefore, results may not be generalizable to trainees not enrolled at academic medical centers. Other potential confounding factors, such as trainees’ cultural values, attitudes, and beliefs, which were not explored in this study, may account for some observed differences in perceived deficits and ratings of cancer pain management training.

Variability in the patient population, resource availability, and affordability at the academic centers surveyed in this study inadvertently influence the trainee experience and are potential confounding factors. It would be instructive to explore how these differences affect trainee clinical competence in cancer pain management. Variability, although a limitation, is a blessing in disguise, because it opens up more avenues for potentially interesting questions that could expand our understanding of the complex interplay between educational environment and competency in cancer pain management. Despite these limitations, the high response rate, large sample size, comprehensive list of targetable deficits, and multicenter approach are important strengths.

One clear implication from this study is that there are unique areas of opportunity to improve cancer pain management education in both high- and low-resource settings. The incorporation of community engagement, cancer pain research, and process and quality improvement in cancer pain management curricula holds the promise to equip trainees with the requisite skills to advance the care of patients with cancer nationally and globally.

## References

[1] Kopf A, Patel NB Guide to pain management in low-resource settings..

[2] Namukwaya E, Leng M, Downing J (2011). Cancer pain management in resource-limited settings: A practice review. Pain Res Treat.

[3] Ogboli-Nwasor E, Makama J, Yusufu L (2013). Evaluation of knowledge of cancer pain management among medical practitioners in a low-resource setting. J Pain Res.

[4] (2008).

[5] Gwyther L, Brennan F, Harding R (2009). Advancing palliative care as a human right. J Pain Symptom Manage.

[6] Connor S, Bermedo MS (2014). Global atlas of palliative care at the end of life.

[7] Gordon DB, Dahl JL, Miaskowski C (2005). American Pain Society recommendations for improving the quality of acute and cancer pain management: American Pain Society Quality of Care Task Force. Arch Intern Med.

[8] Portenoy RK, Lesage P (1999). Management of cancer pain. Lancet.

[9] van den Beuken-van Everdingen MH, de Rijke JM, Kessels AG (2007). Prevalence of pain in patients with cancer: A systematic review of the past 40 years. Ann Oncol.

[10] Breuer B, Fleishman SB, Cruciani RA (2011). Medical oncologists’ attitudes and practice in cancer pain management: A national survey. J Clin Oncol.

[11] Breuer B, Chang VT, Von Roenn JH (2015). How well do medical oncologists manage chronic cancer pain? A national survey. Oncologist.

[12] Deandrea S, Montanari M, Moja L (2008). Prevalence of undertreatment in cancer pain. A review of published literature. Ann Oncol.

[13] Meuser T, Pietruck C, Radbruch L (2001). Symptoms during cancer pain treatment following WHO-guidelines: A longitudinal follow-up study of symptom prevalence, severity and etiology. Pain.

[14] Wood EH (2004). The National Comprehensive Cancer Network (NCCN). J Med Libr Assoc.

[15] Zhao F (2014).

[16] Zech DF, Grond S, Lynch J (1995). Validation of World Health Organization Guidelines for cancer pain relief: A 10-year prospective study. Pain.

[17] Sepúlveda C, Marlin A, Yoshida T (2002). Palliative care: The World Health Organization’s global perspective. J Pain Symptom Manage.

[18] Temel JS, Greer JA, Muzikansky A (2010). Early palliative care for patients with metastatic non-small-cell lung cancer. N Engl J Med.

[19] Haozous E, Doorenbos AZ, Demiris G (2012). Role of telehealth/videoconferencing in managing cancer pain in rural American Indian communities. Psychooncology.

[20] Vargas-Schaffer G (2010). Is the WHO analgesic ladder still valid? Twenty-four years of experience..

[21] Azevedo São Leão Ferreira K, Kimura M, Jacobsen Teixeira M (2006). The WHO analgesic ladder for cancer pain control, twenty years of use. How much pain relief does one get from using it?. Support Care Cancer.

[22] Cherny NI, Cleary J, Scholten W (2013). The Global Opioid Policy Initiative (GOPI) project to evaluate the availability and accessibility of opioids for the management of cancer pain in Africa, Asia, Latin America and the Caribbean, and the Middle East: Introduction and methodology. Ann Oncol.

[23] Cleary J, Silbermann M, Scholten W http://dx.doi.org/10.1093/annonc/mdt503.

[24] Cherny NI, Cleary J, Scholten W http://dx.doi.org/10.1093/annonc/mdt498.

[25] Cleary J, Radbruch L, Torode J (2013). Next steps in access and availability of opioids for the treatment of cancer pain: Reaching the tipping point?. Ann Oncol.

[26] Vega-Stromberg T, Holmes SB, Gorski LA (2002). Road to excellence in pain management: Research, outcomes and direction (ROAD). J Nurs Care Qual.

[27] Elzawawy AM (2015). Could African and low-and middle-income countries contribute scientifically to global cancer care?.

[28] Mellstedt H Cancer initiatives in developing countries..

[29] Govindarajan V, Trimble C (2013).

